# Outcomes Vary by Pre-Operative Physical Activity Levels in Total Knee Arthroplasty Patients

**DOI:** 10.3390/jcm13010125

**Published:** 2023-12-25

**Authors:** Roberta E. Redfern, David A. Crawford, Adolph V. Lombardi, Krishna R. Tripuraneni, David C. Van Andel, Mike B. Anderson, Jason M. Cholewa

**Affiliations:** 1 Clinical Affairs, Zimmer Biomet, Warsaw, IN 46580, USAmike.anderson@zimmerbiomet.com (M.B.A.);; 2 Joint Implant Surgeons, Inc., New Albany, OH 43054, USA; crawfordda@jisortho.com (D.A.C.); lombardiav@jisortho.com (A.V.L.J.); 3 New Mexico Orthopaedics, Albuquerque, NM 87110, USA

**Keywords:** total knee arthroplasty, mobile health, step count, physical activity

## Abstract

Physical activity (PA) is suggested to reduce osteoarthritis pain; however, it may be avoided by patients requiring arthroplasty. Our goal was to investigate objective and patient-reported outcomes as a function of pre-operative PA levels in patients undergoing total knee arthroplasty (TKA). A total of 1941 patients enrolled in a multicenter prospective cohort study investigating a smartphone-based care management platform for self-directed rehabilitation underwent TKA and were included in the analysis. Activity was categorized based on the cohort’s step count quartiles into low, moderate, and high pre-operative PA. Pre-operative and post-operative pain, EQ5D5L, KOOS JR, and step counts were compared by ANOVA according to activity group. Pre-operative pain scores increased with the decreasing activity level (all, *p* < 0.05) and were most improved post-operatively in the low PA group. High PA patients demonstrated the smallest improvements in EQ-5D-5L and KOOS JR. Low and moderate PA patients increased physical activity by three months, reaching 176% and 104% of pre-operative steps; high PA patients did not return to full step counts by one year post-operatively. Patients undergoing TKA who present with higher levels of physical activity report lower levels of pain and higher function pre-operatively but appreciate less improvement up to one year post-operatively. These results may be helpful in appropriate counseling of patient expectations before TKA.

## 1. Introduction

Knee osteoarthritis (OA) affects approximately 654.1 million people over the age of 40 globally [1] and is the fastest-growing driver of disability in developed countries [2]. There now exists considerable evidence to support the inclusion of physical activity in the management of knee OA [3]. Moderate intensity physical activity improves muscular strength, markers of inflammation [4], joint space narrowing [5], pain [6], physical function [7], quality of life [8], and may indirectly improve psychosocial measures by affecting coping mechanisms and self-efficacy [9]. Despite clinician recommendations to exercise [3], knee OA patients tend to perform less physical activity than age-matched controls [10], and in the National Health and Nutrition Examination Survey (NHANES) database, only 9.6% of OA patients accumulated the recommended 150 min of weekly moderate to vigorous physical activity [11]. Unfortunately, activity may be avoided or limited by patients with OA due to increased muscle weakness, pain, psychological distress, and fear-avoidance behaviors [12,13]. Gunn et al. [13] reported 77% of knee OA patients indicated fear of movement, with 58.3% of patients believing that “being careful that I do not make unnecessary movements is the safest thing I can do to prevent pain from worsening” and over 40% expressing fear they may accidentally injure themselves or worsen their pain by engaging in exercise.

The ability to return to physical activity and recreation is a common expectation of total knee arthroplasty (TKA) patients [14]. Multiple systematic reviews and meta-analyses suggest that physical activity recovers within 3 months post-operatively and exceeds pre-operative levels by 6 to 12 months post-operatively [10,15,16]. However, most TKA patients remain less active than age-matched controls through the first year post-operatively [10,16,17]. The importance of physical activity as part of TKA rehabilitation has been well established [18,19]. Appropriate progression of physical activity has been reported to improve objective measures of physical function [20,21,22,23]; however, the relationship between patient-reported outcome measures (PROMs) and post-operative physical activity is currently ambiguous [24]. Several studies suggest a disconnect between PROMs and measures of physical performance, whereby PROMs improve in the first month post-operatively while physical activity initially declines [25,26,27,28,29]. The relationship between PROMs and physical activity after the first three months post-operatively is also ambiguous [24]. Some studies report no [30,31,32] correlation between physical activity and PROMs while others report weak correlations [26,27]. This disconnect between physical activity and PROMs may be due to the relationship between pain and function on PROMs, whereby many items querying physical activity also contain a pain component [33,34], leading patients to conflate reductions in pain for increases in function [35].

Pre-operative exercise interventions have been proposed as a strategy to accelerate the recovery from TKA. Some studies that employ a pre-operative exercise intervention report reduced pain and the accelerated recovery of physical activity or function in the immediate post-operative period [36,37], whereas others suggest this benefit is lost by six months post-operatively [38]. Habitual pre-operative activity may also be related to patient satisfaction and post-operative PROMs. Ponzio et al. [14] reported active TKA patients had higher expectations to return to physical recreation activities than inactive patients; however, there were no differences in satisfaction or PROMs between groups at two years post-operatively. On the other hand, significantly more inactive patients increased their activity above pre-operative levels and less inactive patients reported a reduction in activity values at two years post-operatively [14]. While the relationship between pre-operative PROMs and post-operative outcomes suggests patients with higher pre-operative PROMs are likely to experience less improvements [39,40], the relationship between habitual physical activity and post-operative outcomes requires further study. Our goal was to investigate objective and patient-reported outcomes as a function of pre-operative PA levels in patients undergoing total knee arthroplasty (TKA).

## 2. Materials and Methods

A secondary analysis of a multicenter prospective longitudinal cohort study was performed. Patients at least 18 years of age and undergoing TKA for treatment of end-stage osteoarthritis were assessed for eligibility in a trial investigating the use of a smartphone-based care management platform with smartwatch (mymobility^®^, Zimmer Biomet, Warsaw, IN, USA) for self-directed rehabilitation following arthroplasty (Clinicaltrials.gov identifier NCT03737149). The application provides arthroplasty-specific education pre- and post-operatively, as well as video-guided exercises and delivery of appropriate PROMs questionnaires at specified time intervals up to one year following surgery. All patients were required to own an Apple iPhone^®^ (Apple Inc., Cupertino, CA, USA) capable of pairing with the Apple Watch^®^ (Apple Inc., Cupertino, CA, USA), which was provided to all enrolled participants. Eligibility requirements also included ambulation with no more than a single crutch or cane pre-operatively. Participants were excluded if undergoing simultaneous or staged (<90 days) bilateral procedures, participating in any other surgical intervention, physical therapy, or pain management studies, or if deemed to be a current drug or alcohol abuser. Approval by a Central IRB was obtained prior to study commencement. All participants provided written informed consent upon enrollment. A total of 1941 patients who underwent TKA at 28 sites between November 2018 and November 2022 were eligible to be included in this analysis and volunteered to participate.

Participants downloaded the application at least two weeks prior to surgical intervention, allowing for the passive collection of pre-operative activity (daily step counts). Objective mobility metrics were collected by the application for up to 425 days post-operatively or until study exit or withdrawal. Average daily step counts were calculated over the two-week pre-operative period and over a one-week period centered at each interval in the post-operative periods. Post-operative activity was only included if data were provided in at least four of seven days in these periods. Patients completed general and joint-specific PROMs including EQ-5D-5L, EQ-VAS, and Knee Injury and Osteoarthritis Outcomes—Joint Replacement (KOOS JR) pre-operatively through one year post-operatively. The KOOS JR is a validated instrument for the evaluation of knee replacement outcomes combining pain, symptoms, and functional limitations ranging from 0 to 100 points, where 0 indicates the worst level of pain and function and 100 reflects perfect joint health. The pain numeric rating score (NRS, 0–10 points) and Knee Society Score (KSS) satisfaction subscale (0–40 points) were completed pre-operatively and at one and three months post-operatively.

Average daily step counts were assessed over the entire cohort to categorize patients’ relative activity levels prior to surgery, where those in the bottom quartile were labeled as low, the middle two quartiles (25th–75th percentile) were labeled moderate, and the top quartile categorized as high activity. Baseline characteristics and post-operative outcomes including PROMs, satisfaction, pain, and step counts were compared between activity categories by one-way ANOVA with ad-hoc Tukey pairwise comparisons. Changes from baseline for all outcome measures were also compared in this manner, as were the percentage of steps performed through one year compared to pre-operatively. Paired t tests were used to investigate changes in step counts for each individual pre-operative activity level. Continuous variables are presented as mean ± SD; categorical data are presented as counts and percent and compared via chi-square analysis. All analyses were performed with SAS Enterprise Guide v7.1 (2014 SAS Institute Inc., Cary, NC, USA); *p* values < 0.05 were considered statistically significant.

## 3. Results

The average age over the entire cohort was 64.5 ± 8.9 years. In total, 60.7% were female with an average BMI of 31.4 ± 6.3 (Table 1). The median pre-operative step count in all participants was 5211 steps per day (IQR 3086–6765, Table 2). The average daily step counts in the low-, moderate-, and high-activity groups were 2025, 4817, and 9180, respectively. Age and BMI varied over activity groups, where the lower-activity groups were older with a higher BMI. There was also a preponderance of females (70.8%) in the low-activity group (*p* < 0.0001).

**Figure 1:**
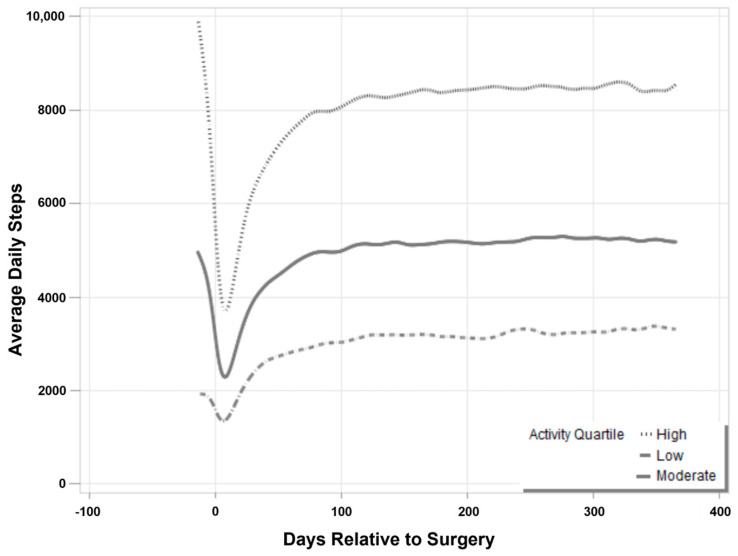
Step count recovery curves as a function of baseline activity.

Average daily steps at specified intervals through one year post-operatively were compared between pre-operative activity groups (Table 2). Participants who performed the highest quartile of steps prior to TKA continued to perform more steps through every period investigated. However, considering the recovery of step counts as a percentage of the average performed before surgery, the participants categorized as the low-activity group exceeded their pre-operative steps by one month, performing 145% of their baseline activity, while both moderate- and high-activity patients did not yet achieve this milestone. This trend continued at six weeks post-operatively, where low pre-operative activity participants continued to improve and exceed baseline step counts, while moderate- and high-activity participants did not yet reach full pre-operative levels. On average, 3058, 4948, and 7949 steps were performed daily at three months following TKA in those who had been categorized as low, moderate, and high activity prior to surgery, respectively. Low pre-operative PA participants returned to 177% of their pre-operative steps at three months, moderate-activity participants returned to 104% of average daily steps, and the high-activity group did not reach pre-operative levels, performing 88% of their pre-operative average steps (Figure 1). While the low- and moderate-activity groups both exceeded pre-operative activity levels by three months post-operatively, those with the highest pre-operative counts did not achieve this goal at one year, reaching 94.6% of their pre-operative levels.

Paired t tests at each of these time points indicated that the change in the low pre-operative activity participant’s step counts reached statistically, though not clinically, significant improvement by four weeks post-operatively (Table 3). Moderate-activity participants did not significantly improve step counts until six months, while highly active participants continued to demonstrate significantly reduced step counts by an average of over 600 steps at one year post-operatively.

Knee function as measured by the KOOS JR varied pre-operatively, though the differences between groups do not meet a previously reported minimal clinically important difference (MCID) [41]. The lowest knee function as measured by the KOOS JR was reported by the low-activity group, increasing among higher-activity level participants (49.3 vs. 51.5 vs. 54.4, *p* < 0.0001, all pairwise comparisons *p* < 0.05). KOOS JR scores did not vary between groups through six months following procedures, with significant, though not clinically important, differences at one year post-operatively (Table 4). Those in the low PA group reported greater improvements over baseline through three months compared to both other groups. Pairwise comparisons revealed that the change in KOOS JR scores was lowest in the high-activity group with greater improvement observed in both the low- and moderate-activity participants.

Pre-operative pain scores varied by activity group, with the highest pain reported in the low-activity group (5.95) and lowest pain in the high-activity group (5.28, *p* < 0.0001, all pairwise comparisons *p* < 0.05). At one month following surgery, pain was highest in the low PA group, varying significantly from both the moderate and high PA participants (Table 5). However, reduction in pain at this time was also largest in those with low pre-operative activity, differing only from the high PA group on pairwise comparison. At three months post-operatively, pain was similar between the activity groups, with a larger reduction from baseline observed only between the high- and low-activity participants (−2.59 vs. −3.15, *p* < 0.05).

General health PROMs varied in a similar manner by pre-operative activity. Low-activity participants reported the lowest health-related quality of life on the EQ-5D-5L and EQ-VAS (Table 5). EQ-5D-5L scores continued to vary by groups throughout each post-operative interval. The change in score from baseline was the highest in the low-activity group, with all pairwise comparisons significant at one month, and both low and moderate groups demonstrating larger improvements than the high PA group at one year. The general rating of overall health as reported on EQ-VAS was similar among groups at one month post-operatively, where changes from baseline were greatest in the low PA group and showed evidence of decline in those with high PA before TKA. At one year, participants with low PA demonstrated the lowest scores, differing only from the high PA group, and only the lowest-activity group reported significantly greater improvements at this timepoint. Pre-operative knee satisfaction varied between all groups on the KSS satisfaction subscale; these scores varied only between the low and high groups at one month but did not vary amongst activity groups at three months, nor did score changes from baseline at either point (Table 5, *p* = 0.051).

## 4. Discussion

In this analysis of a large prospective cohort of participants in which physical activity was objectively measured in patients undergoing TKA both pre- and post-operatively, large variations in pre-operative activity levels were observed. Investigation of recovery as a function of activity prior to arthroplasty demonstrated that participants with the lowest level of PA before surgery recovered step counts by one month post-procedurally, performing nearly 1000 steps more than before TKA by three months, reaching and maintaining approximately 180% of pre-operative steps through one year post-operatively. Participants in the two middle quartiles of pre-operative step counts recovered activity with a modest increase in step counts one year after surgery, while those in the highest quartile of activity did not return to pre-operative levels at any interval through one year. The lowest-activity participants reported greater improvements in joint function, pain, and health-related quality of life. Despite this, satisfaction scores did not vary between groups.

Use of accelerometry and wearable activity monitors for the collection of objective mobility data is becoming more common as an adjunct to traditional methods for the monitoring of recovery following surgery. Previous studies of activity relied upon self-reporting by patients utilizing questionnaires or activity diaries. These methods have often demonstrated perceived increases in physical activity by patients after TKA. However, research utilizing both subjective and objective measures of PA have indicated that objective measures do not correlate strongly with subjective reports [27,42]. A study of test–retest reliability found higher reliability for activity measurement with the use of an armband than actigraphy or questionnaires, suggesting self-reporting excludes incidental movement [43]. Walker et al. found that patients increased activity by 79% at six months but reported reduced mobility [44], while de Groot et al. observed patients’ reporting of physical activity via a validated questionnaire was significantly larger than their actual activity as measured by accelerometry, finding only a 0.7% mean increase in movement-related activity [45]. Similarly, patients asked to estimate the distance they walked while blinded to the readings of wearables revealed a 69% mean error pre-operatively, which increased over time to 93% post-operatively [46]. Importantly, a minority of patients in that study were consistently under- or over-estimators, and subjective reports of activity increased temporally but actual distance walked decreased. Researchers have hypothesized that participants frequently over-estimate the amount of physical activity performed after TKA due to improvements in function and pain, noting that better function does not necessarily translate to increased activity, though perceived pain appears to be linked to perceived PA [42,47].

Our data agree with several reports in the literature regarding the amount of physical activity performed by patients prior to TKA. A previous review of the literature to define average steps per day in special populations reported 3500–5500 steps per day in adults with chronic illness, where those with osteoarthritis averaged 4086 and OA with arthroplasty 4892 [48]. However, studies of the change in objective activity following TKA have provided conflicting results. While authors suggest that patients typically return to pre-operative step counts by six to nine weeks [27,49], reports have differed with regard to whether activity increases significantly after TKA. Some studies have reported significant increases at six months or one year in low-to-moderately active participants [50,51]. These studies have found increases generally of about 1000 additional steps per day, similar to our observation in the low- and moderate-activity participants in this study. Other studies using activity monitors have suggested no significant change in PA after arthroplasty [16,52]. Harding et al. reported this trend in terms of daily activity counts, with insignificantly decreased counts at 6 months following TKA [53]. Investigations reporting activity in terms of the percentage of time spent in sedentary or moderate to vigorous physical activity (MVPA) have observed similar trends [54]. Multiple studies conclude that the majority of patients do not adopt more active lifestyles even up to four years after surgery [45,55,56].

It is possible that one source of variance in the conclusions of these studies is due to the populations involved. Considering all arthroplasty patients without considering pre-operative activity levels, our data would suggest a similar trend, as the entire cohort performed 5211 steps pre-operatively and increased to only 5730 steps per day post-operatively. Of note, while the low-activity participants did increase activity, they did not appear to adopt active lifestyles after TKA. Other studies have noted observations similar to ours, using both subjective and objective measures. Patients who were considered high activity prior to their procedures reported a decrease in physical activity, while those considered low activity pre-operatively reported increased physical activity post-TKA via the Lower Extremity Activity Scale (LEAS) [14,57]. Lutzner et al. also found that patients who were considered higher activity (performing more than 5000 steps daily) before TKA only improved 8.2% compared to a 59.6% improvement in those who walked less pre-operatively [58]. While pre-operative step counts appear to be a strong predictor of post-operative steps, and higher-activity patients do continue to perform more activity after TKA, these data may be helpful in counseling patient expectations, particularly in younger active patients undergoing arthroplasty to maintain active lifestyles. It is important to note that both the low and high baseline activity groups remained within their pre-operative lifestyle groups following intervention through one year [48]. Despite significant increases in daily step counts, those in the low baseline PA group remained sedentary post-operatively (<5000 steps per day). High baseline activity patients also remained within the somewhat active lifestyle category (7500–9000 steps per day). Only those in the moderate-activity group improved on the whole from sedentary to low-active (5000–7499 steps per day) [48]. These results suggest that not only should surgeons indicate to high-activity patients that returning to their full activity may be gradual, and they may not exceed that activity within the first year post-operatively, but appropriate counseling of low-activity patients is also warranted. Though these patients may expect to exceed pre-operative PA more quickly, daily step goals to achieve a non-sedentary lifestyle should be discussed pre- and post-operatively. In particular, surgeons may provide reassurance that increased activity will not result in reduced survivorship of the implant and will improve healing, empowering patients to be engaged and active throughout their recovery.

Previous studies have noted that PROMs do not reliably assess return to function, and alternate means should be used to monitor this outcome [27]. Our data support these assertions; while pre-operative KOOS JR scores varied in a statistically significant manner, the largest difference between groups was approximately 5 points, suggesting no clinically important difference based on established MCID thresholds (6–14 points) [41]. Similarly, while low-activity participants significantly increased activity after surgery and high-activity participants experienced a decrease in step counts, both reported significant improvements on KOOS JR by three months. While the low-activity group reported the largest improvement, the difference in change from baseline between the low and high groups (4.14 points) did not meet clinical significance. These results highlight previous suggestions in the literature regarding the role of objectively measured activity as a complementary method to assess post-arthroplasty recovery.

It is of interest that the lower-activity participants reported higher levels of pain pre-operatively, though not completely unexpected. However, others have asserted that higher step counts in osteoarthritis patients contribute to higher reports of pain [59]. The finding that these participants also reported greater reductions in pain could be related to their lower activity following surgery. Patterson et al. suggested that patients who approach activity following arthroplasty with more moderate activity and “take it easy” reported less pain and a better, more consistent overall recovery [60]. Additionally, we are unaware of any studies that have compared general health-related quality-of-life outcomes as a function of pre-operative activity levels. Previous studies have found an association between increased percentage of time in PA and improvements in both general and joint-specific PROMs [42]. While we did not attempt to correlate the changes in quality of life with changes in PA, it is interesting to note that those with lower pre-operative activity reported lower ratings of health and appreciated larger improvements after TKA, with both low- and moderate-activity groups meeting MCID for the EQ-5D-5L and EQ-VAS [61] earlier than high-activity participants. It is also of interest to note that the participants in the highest activity quartile did not report lower satisfaction at three months, particularly given that this group had not yet reached 90% of their previous activity levels at this interval, though change in satisfaction from baseline did trend toward significance at this time.

Our study is strengthened by the relative size of the cohort included and prospective nature of the data collection but is also subject to limitations. As a multicenter trial, it was not possible to standardize and account for patient counseling of expectations, which may have impacted patients’ approaches to activity after surgery or their expectations and thus satisfaction. Our relatively short pre-operative period of evaluation and follow-up could also impact results, as we only collected pain and satisfaction data for three months following surgery. It is unclear whether activity recovery may have continued to progress more than one year after surgery, particularly in the high-activity group who had not yet plateaued at this time. While the literature investigating changes in PROMs and subjective reports of activity find no substantial changes beyond one year post-operatively [62], it is less clear whether this is true for objective activity data. Moreover, we measured only the volume of step counts, rather than investigating the intensity of activity or types of additional activity that may have been undertaken by participants, such as swimming, bicycling, or strength training exercises, which may have been more common in the higher-activity groups. Thus, it is unclear from these data whether patients were able to fully return to all their pre-operative activities, though contraindicated activities may vary per patient and surgeon after TKA. In addition, all included participants were required to own a smartphone for inclusion. While this has become nearly ubiquitous in some segments of the population, not all older adults possess this technology and thus our results may not be generalizable to this population. Patients who consented to participate in a study of this platform for self-directed rehabilitation may have been more motivated during post-operative recovery than the general population, which could limit generalizability to the knee arthroplasty population at-large. Previous studies have also suggested that patients receiving feedback from wearables regarding daily PA perform more steps daily. It is possible that this may have affected pre-operative activity in this cohort, where participants may have performed more PA than would have been performed habitually without the use of the activity trackers. The availability of feedback from the trackers in the post-operative period may also have impacted PA levels such that our results may not be generalizable to patients not utilizing wearables for recovery monitoring after TKA. Finally, the ability to compare our findings to previous reports in the literature are limited by the heterogeneity of methods to collect objective mobility data, as well as the variety of methods used to define low, moderate, and high activity in OA and arthroplasty populations.

## 5. Conclusions

Patients who perform high levels of activity prior to TKA may not fully recover to pre-operative levels within one year of surgery. Though these patients may express higher levels of activity overall, they may not achieve the same level of benefit with regard to pain reduction or improvement in general quality of life. These findings can be incorporated into conversations regarding post-operative expectations, particularly in younger patients who undergo TKA to maintain an active lifestyle. Patients with low levels of activity prior to TKA should be counseled regarding the benefits of physical activity, made aware of their sedentary status, and encouraged to increase physical activity following surgery to enhance the benefits of the procedure.

## Figures and Tables

**Table 1:** Baseline patient characteristics.

	All Activity Levels	Low Pre-Operative Activity	Moderate Pre-Operative Activity	High Pre-Operative Activity	*p* Value
Age	64.5 ± 8.9	66.4 ± 8.8	64.2 ± 9.0	63.2 ± 8.5	<0.0001
BMI	31.4 ± 6.3	33.1 ± 6.7	31.8 ± 6.2	29.0 ± 5.5	<0.0001
Sex—female	1178 (60.7)	344 (70.8)	577 (59.6)	257 (52.9)	<0.0001

**Table 2:** Average step counts over cohort and by pre-operative activity levels.

	All Activity Levels	Low Pre-Operative Activity	Moderate Pre-Operative Activity	High Pre-Operative Activity	*p* Value
Pre-operative step count	5211 ± 2943	2025 ± 726	4817 ± 1034	9180 ± 2392	<0.0001
4 weeks	4144 ± 2442	2356 ± 1523	3931 ± 1851	6277 ± 2617	<0.0001
6 weeks	4601 ± 2538	2679 ± 1647	4345 ± 1862	6937 ± 2631	<0.0001
3 months	5280 ± 2880	3058 ± 1910	4948 ± 2079	7949 ± 2911	<0.0001
6 months	5529 ± 3126	3071 ± 2074	5186 ± 2295	8378 ± 3106	<0.0001
12 months	5730 ± 3390	3281 ± 2481	5225 ± 2522	8749 ± 3341	<0.0001
Percent recovery					
4 weeks	94.4 ± 133.7	145.1 ± 258.4	82.6 ± 39.2	69.7 ± 27.1	<0.0001
6 weeks	105.1 ± 128.7	164.3 ± 246.5	91.3 ± 40.0	77.1 ± 40.0	<0.0001
3 months	116.9 ± 129.3	176.7 ± 247.3	104.0 ± 43.8	87.7 ± 29.4	<0.0001
6 months	121.6 ± 147.9	181.3 ± 287.7	109.5 ± 49.8	92.0 ± 32.9	<0.0001
12 months	121.3 ± 88.4	175.2 ± 154	108.6 ± 51.2	94.6 ± 32.4	<0.0001

**Table 3:** Paired t tests indicating change in steps from pre-operative to each follow-up period.

Step Count Change from Pre-Operative	Low Pre-Operative Activity	*p* Value	Moderate Pre-Operative Activity	*p* Value	High Pre-Operative Activity	*p* Value
4 weeks	306 ± 1586	<0.0001	−907 ± 1838	<0.0001	−2902 ± 2885	<0.0001
6 weeks	632 ± 1698	<0.0001	−491 ± 1811	<0.0001	−2245 ± 2864	<0.0001
3 months	966 ± 1916	<0.0001	125 ± 1954	0.07	−1277 ± 2880	<0.0001
6 months	1016 ± 2071	<0.0001	365 ± 2221	<0.0001	−938 ± 3108	<0.0001
12 months	1213 ± 2424	<0.0001	399 ± 2399	0.0001	−653 ± 3040	0.0003

**Table 4:** KOOS JR scores and changes (Δ) from baseline in the overall cohort and by pre-operative activity levels.

KOOS JR	All Activity Levels	Low Pre-Operative Activity	Moderate Pre-Operative Activity	High Pre-Operative Activity	*p* Value
Pre-operative	51.71 ± 12.20	49.26 ± 12.53	51.54 ± 11.86	54.38 ± 12.02	<0.0001
1 month	63.03 ± 10.28	62.67 ± 11.11	62.97 ± 10.31	63.51 ± 9.34	0.45
3 months	70.36 ± 12.03	69.97 ± 11.68	70.31 ± 12.28	70.80 ± 11.85	0.60
6 months	74.61 ± 13.49	73.56 ± 13.49	74.65 ± 13.97	75.53 ± 12.54	0.12
12 months	80.32 ± 14.20	78.38 ± 15.34	81.43 ± 13.55	79.90 ± 14.23	0.01
Δ 1 month	11.29 ± 13.78	13.27 ± 14.51	11.42 ± 13.82	9.15 ± 12.66	<0.0001
Δ 3 months	18.48 ± 14.86	20.17 ± 14.23	18.80 ± 15.15	16.38 ± 14.64	0.0008
Δ 6 months	22.83 ± 15.67	23.67 ± 15.96	23.19 ± 15.75	21.34 ± 15.15	0.08
Δ 12 months	28.70 ± 16.96	29.7 ± 17.84	29.89 ± 16.67	25.56 ± 16.37	0.0007

**Table 5:** General and health-related quality-of-life patient-reported outcome measures with changes (Δ) from baseline.

	All Activity Levels	Low Pre-Operative Activity	Moderate Pre-Operative Activity	High Pre-Operative Activity	*p* Value
Pain					
Pre-operative	5.62 ± 2.08	5.95 ± 1.96	5.63 ± 2.11	5.28 ± 2.07	<0.0001
30 days	3.82 ± 2.03	4.10 ± 2.14	3.73 ± 2.02	3.74 ± 1.93	0.01
90 days	2.65 ± 2.12	2.80 ± 2.26	2.56 ± 2.08	2.68 ± 2.04	0.18
Δ 30 days	−1.73 ± 2.48	−1.92 ± 2.45	−1.80 ± 2.57	−1.45 ± 2.33	0.02
Δ 90 days	−2.89 ± 2.63	−3.15 ± 2.50	−2.94 ± 2.71	−2.59 ± 2.58	0.01
KSS Satisfaction					
Pre-operative	13.4 ± 7.6	12.1 ± 7.2	13.2 ± 7.6	14.7 ± 7.8	<0.0001
30 days	23.6 ± 11.0	22.4 ± 11.6	23.8 ± 11.0	24.4 ± 10.0	0.02
90 days	28.8 ± 9.5	27.9 ± 10.0	29.0 ± 9.4	29.2 ± 9.2	0.09
Δ 30 days	10.7 ± 12.3	11.2 ± 12.5	11.1 ± 12.4	9.7 ± 11.7	0.11
Δ 90 days	15.2 ± 11.3	15.6 ± 11.3	15.6 ± 11.7	14.0 ± 10.7	0.051
EQ-5D-5L					
Pre-operative	0.572 ± 0.250	0.506 ± 0.281	0.569 ± 0.242	0.638 ± 0.214	<0.0001
30 days	0.689 ± 0.191	0.668 ± 0.196	0.695 ± 0.192	0.699 ± 0.183	0.03
90 days EQ-5D-5L	0.803 ± 0.167	0.781 ± 0.168	0.808 ± 0.168	0.812 ± 0.162	0.02
6 months	0.833 ± 0.176	0.812 ± 0.195	0.832 ± 0.176	0.853 ± 0.154	0.007
12 months	0.864 ± 0.169	0.828 ± 0.216	0.872 ± 0.156	0.879 ± 0.139	0.0003
Δ 30 days	0.112 ± 0.262	0.165 ± 0.261	0.118 ± 0.268	0.053 ± 0.243	<0.0001
Δ 90 days	0.223 ± 0.248	0.269 ± 0.247	0.231 ± 0.256	0.170 ± 0.224	<0.0001
Δ 6 months	0.256 ± 0.256	0.305 ± 0.265	0.258 ± 0.261	0.211 ± 0.229	<0.0001
Δ 12 months	0.281 ± 0.236	0.328 ± 0.250	0.289 ± 0.241	0.228 ± 0.199	<0.0001
EQ-VAS					
Pre-operative	75.46 ± 15.7	71.53 ± 17.2	75.72 ± 15.5	78.72 ± 13.7	<0.0001
30 days	77.22 ± 14.64	76.22 ± 14.65	77.38 ± 14.77	77.84 ± 14.38	0.26
90 days	82.6 ± 12.6	80.8 ± 13.4	82.8 ± 12.6	83.8 ± 11.7	0.004
6 months	83.67 ± 12.46	81.65 ± 14.28	83.48 ± 12.17	85.79 ± 10.85	<0.0001
12 months	85.09 ± 12.49	82.94 ± 15.19	85.06 ± 11.45	87.0 ± 11.58	0.0005
Δ 30 days	1.65 ± 16.54	5.12 ± 17.19	1.60 ± 16.10	−1.31 ± 16.23	<0.0001
Δ 90 days	6.89 ± 15.5	9.22 ± 16.1	6.75 ± 15.5	5.18 ± 14.9	0.002
Δ 6 months	8.11 ± 15.32	11.01 ± 18.03	7.45 ± 14.84	6.84 ± 13.23	0.0007
Δ 12 months	8.87 ± 15.48	10.73 ± 17.98	8.81 ± 14.36	7.46 ± 15.24	0.05

## Data Availability

The data presented in this study are available on request from the corresponding author. The data are not publicly available due to privacy restrictions.
